# Blood Fluke Exploitation of Non-Cognate CD4^+^ T Cell Help to Facilitate Parasite Development

**DOI:** 10.1371/journal.ppat.1000892

**Published:** 2010-04-29

**Authors:** Erika W. Lamb, Colleen D. Walls, John T. Pesce, Diana K. Riner, Sean K. Maynard, Emily T. Crow, Thomas A. Wynn, Brian C. Schaefer, Stephen J. Davies

**Affiliations:** 1 Department of Microbiology and Immunology, Uniformed Services University of the Health Sciences, Bethesda, Maryland, United States of America; 2 Laboratory of Parasitic Diseases, National Institute of Allergy and Infectious Diseases, National Institutes of Health, Rockville, Maryland, United States of America; Trudeau Institute, United States of America

## Abstract

*Schistosoma* blood flukes, which infect over 200 million people globally, co-opt CD4^+^ T cell-dependent mechanisms to facilitate parasite development and egg excretion. The latter requires Th2 responses, while the mechanism underpinning the former has remained obscure. Using mice that are either defective in T cell receptor (TCR) signaling or that lack TCRs that can respond to schistosomes, we show that naïve CD4^+^ T cells facilitate schistosome development in the absence of T cell receptor signaling. Concurrently, the presence of naïve CD4^+^ T cells correlates with both steady-state changes in the expression of genes that are critical for the development of monocytes and macrophages and with significant changes in the composition of peripheral mononuclear phagocyte populations. Finally, we show that direct stimulation of the mononuclear phagocyte system restores blood fluke development in the absence of CD4^+^ T cells. Thus we conclude that schistosomes co-opt innate immune signals to facilitate their development and that the role of CD4^+^ T cells in this process may be limited to the provision of non-cognate help for mononuclear phagocyte function. Our findings have significance for understanding interactions between schistosomiasis and other co-infections, such as bacterial infections and human immunodeficiency virus infection, which potently stimulate innate responses or interfere with T cell help, respectively. An understanding of immunological factors that either promote or inhibit schistosome development may be valuable in guiding the development of efficacious new therapies and vaccines for schistosomiasis.

## Author Summary

Schistosomes, or blood flukes, cause a debilitating illness in millions of people worldwide, which manifests when inflammation develops in response to parasite eggs that become trapped in the liver and other organs. Paradoxically, schistosomes require signals from the host's immune system in order to develop fully into egg-producing adults. Previously, we showed that CD4^+^ T cells facilitate schistosome development. Here, we show that the mere presence of CD4^+^ T cells is sufficient for schistosome development to proceed. There is no requirement for these cells to respond to the parasite, or to exhibit any typical “effector” response. Two pieces of data suggest this effect on parasite development is mediated by antigen-presenting cells of the innate immune system such as monocytes and macrophages, which interact with CD4^+^ T cells by expressing MHC class II molecules. First, the presence of naïve CD4^+^ T cells correlates with baseline changes in the development of monocyte/macrophage populations. Second, direct stimulation of the monocyte-macrophage system restores parasite development, bypassing the requirement for CD4^+^ T cells in schistosome development. Understanding the mechanisms that promote or inhibit blood fluke infection may facilitate the development of new treatments and vaccines for schistosomiasis.

## Introduction

Extensive co-evolution of parasitic organisms and their hosts has given rise to complex host-parasite relationships in which exploitation of host responses to infection by parasites is a recurring theme. Nowhere is this complexity in host-parasite relationships better exemplified than in the parasitic helminths, which infect a third of the world's human population [1] by establishing chronic infections that persist for years, often in the face of vigorous immune responses [2]. *Schistosoma* blood flukes account for a significant proportion of these helminth infections, causing considerable morbidity and mortality. After initiating infection by direct skin penetration, schistosomes migrate in the bloodstream to hepatic pre-sinusoidal venules, where rapid growth and development ensues, culminating in mating of adult worms and the production of eggs.

It was previously shown that *Schistosoma mansoni* co-opts CD4^+^ T cell-dependent mechanisms to facilitate both parasite development during pre-patent infection and the excretion of parasite eggs after the onset of oviposition [3], [4], [5]. The latter requires formation of Th2-dependent granulomas in the bowel wall to allow passage of eggs from the portal vasculature into the intestinal lumen [3], [4], [6]. However, the mechanism by which CD4^+^ T cells facilitate development of schistosome worms has not been elucidated. While homeostatic maintenance of peripheral CD4^+^ T cells through the action of γ_c_ cytokines is required to provide a permissive environment for schistosome development [7], previous studies did not identify a role for effector Th1/Th2 responses or any single effector cytokine in parasite development [5], [8], [9].

In this study we tested whether activation of CD4^+^ T cells through the T cell receptor (TCR) by schistosome antigens is required for schistosome development to proceed. Unexpectedly, our data indicate that CD4^+^ T cells that lack specificity for schistosome antigens can facilitate schistosome development in the absence of antigen-mediated T cell activation. Interestingly, the presence of naïve, resting CD4^+^ T cells also correlates both with steady-state transcriptional changes in the expression of genes that are critical for development of monocytes and macrophages and with changes in the composition of peripheral monocyte populations. Further, direct stimulation of the mononuclear phagocyte system bypasses the requirement for CD4^+^ T cells in schistosome development, suggesting that innate cells of the mononuclear phagocyte system facilitate schistosome development and that CD4^+^ T cells influence the parasites indirectly by modulating monocyte/macrophage function. Together, our results demonstrate that blood flukes exploit innate immune signals to facilitate their development and suggest that the role of CD4^+^ T cells in this process may be limited to the provision of non-cognate help for innate mononuclear cell function.

## Results

### TCR signaling blockade does not impair parasite development

**Figure 1:**
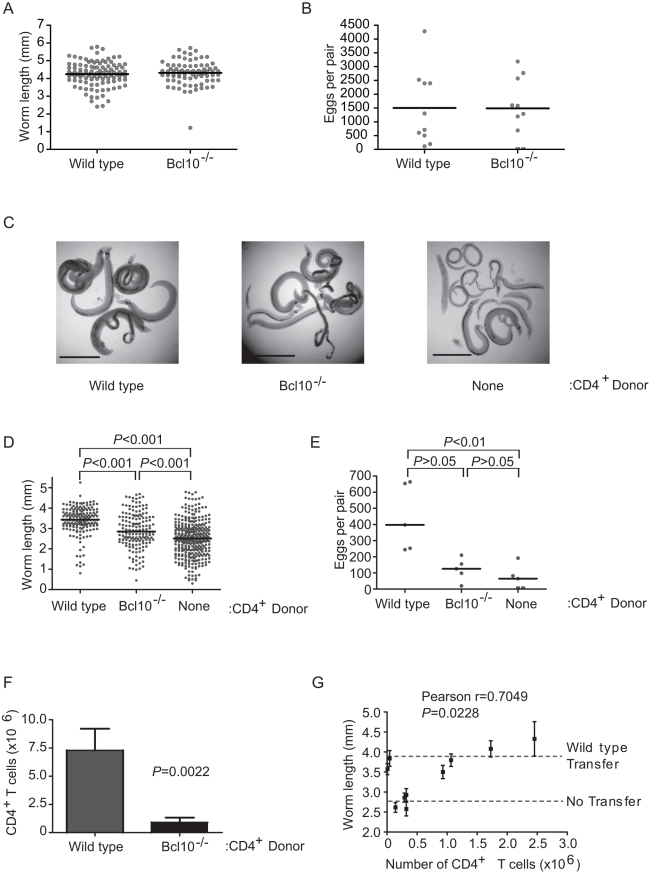
TCR signaling in CD4^+^ T cells is dispensable for *S. mansoni* development. (A) Wild type and Bcl10^-/-^ mice were infected with *S. mansoni*, parasites were recovered from the portal tract at 6 weeks post infection and male worms were measured from digital micrographs. Mean values are represented by horizontal bars. (B) Egg production by schistosome pairs in wild type and Bcl10^-/-^ mice at 6 weeks post infection. Mean values are represented by horizontal bars. (C) RAG-1^-/-^ mice reconstituted with wild type or Bcl10^-/-^ CD4^+^ T cells mice were infected with *S. mansoni* and parasites were recovered from the portal tract at 6 weeks post infection. Representative micrographs of parasites recovered from non-reconstituted RAG-1^-/-^ mice and RAG-1^-/-^ mice reconstituted with wild type or Bcl10^-/-^ CD4^+^ T cells are shown. Bar = 1 mm. (D) Lengths of male worms recovered from reconstituted RAG-1^-/-^ mice described in (C) were measured from digital micrographs. Mean values are represented by horizontal bars. (E) Egg production by schistosome pairs in reconstituted RAG-1^-/-^ mice described in (C) at 6 weeks post infection. Mean values are represented by horizontal bars. (F) At necropsy, the numbers of CD4^+^ T cells in the spleens of RAG-1^-/-^ recipients were determined by flow cytometry. Data are represented as mean +/− SEM. (G) Correlation of average male worm length and CD4^+^ T cell numbers for each RAG-1^-/-^ recipient of Bcl10^-/-^ CD4^+^ T cells. “WT Transfer” and “No Transfer” dashed lines represent the average length of parasites recovered from control mice that received wild type CD4^+^ T cells or PBS alone, respectively. Worm length data for each mouse are represented as mean +/− SEM. Groups of 4–5 mice were used for each experimental condition. Data are representative of three independent experiments.

To evaluate the role of T cell activation in schistosome development, we first examined schistosome development in Bcl10 (Unigene accession no. Mm.239141) -deficient and protein kinase C θ (PKCθ; Mm.329993) –deficient mice, where impairment of NF-κB activation in response to TCR ligation renders T cells unresponsive to antigen [10], [11]. Unexpectedly, *S. mansoni* recovered from Bcl10^-/-^ and wild type mice were indistinguishable in size (Figure 1A) and deposited comparable numbers of eggs (Figure 1B), unlike schistosomes from recombination activating gene 1 (RAG-1; Mm.828) -deficient mice that exhibit a severely stunted phenotype and greatly reduced rates of egg production ([5] and data not shown; see also Figure 1D and E). As expected, CD4^+^ T cell responses to *S. mansoni* were impaired by Bcl10 deletion, as measured by acquisition of an activated phenotype (Figures S1A and B) and cytokine production (Figure S1C), suggesting that activation of CD4^+^ T cells through the TCR and subsequent CD4^+^ T cell responses are dispensable for normal schistosome development. Identical results were obtained with PKCθ^-/-^ mice (data not shown). To directly test whether Bcl10^-/-^ CD4^+^ T cells can facilitate schistosome development, parasite development was examined in RAG-1^-/-^ mice that were reconstituted with Bcl10^-/-^ CD4^+^ T cells. Adoptive transfer of Bcl10^-/-^ CD4^+^ T cells prior to infection partially restored worm growth (Figures 1C and D), resulting in a phenotype that was intermediate between RAG-1^-/-^ recipients of wild type CD4^+^ T cells and non-reconstituted RAG-1^-/-^ mice. Likewise, adoptive transfer of Bcl10^-/-^ CD4^+^ T cells produced an intermediate egg production phenotype that was not significantly different from RAG-1^-/-^ recipients of wild type CD4^+^ T cells or non-reconstituted RAG-1^-/-^ mice, even though egg production in these latter two groups was significantly different from each other (Figure 1E). Identical results were obtained when adoptive transfers were performed with PKCθ^-/-^ CD4^+^ T cells (data not shown). Because TCR signals are required for T cell homeostasis [12], Bcl10^-/-^ CD4^+^ T cells exhibited variable levels of engraftment that were consistently lower than those of wild type cells (Figure 1F). However, there was a significant positive correlation between the number of Bcl10^-/-^ CD4^+^ T cells and the mean length of the worms recovered from each recipient (Figure 1G). These data suggest that CD4^+^ T cells do not require intact antigen receptor signaling to facilitate parasite development. Further, our data suggest there is a simple requirement for sufficient numbers of CD4^+^ T cells to allow parasite development to proceed and predict a minimum of approximately 2×10^6^ CD4^+^ T cells as the number required to restore parasite growth to wild type levels (Figure 1G).

### Non-responsive CD4^+^ T cells facilitate *S. mansoni* development

**Figure 2:**
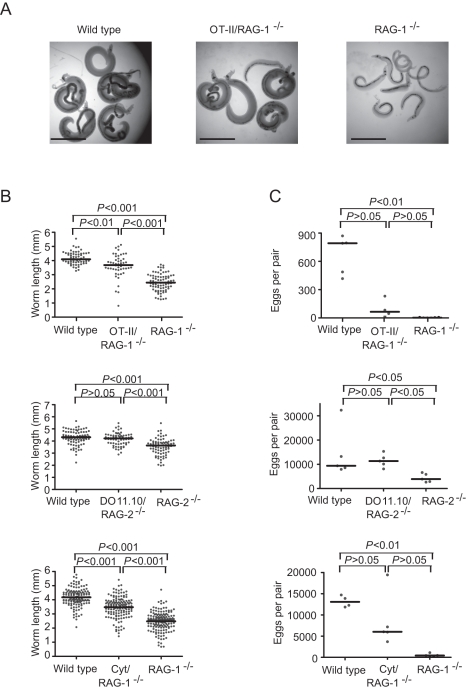
Recognition of schistosome antigens by CD4^+^ T cells is dispensable for *S. mansoni* development. (A) Wild type, OT-II/RAG-1^-/-^ and RAG-1^-/-^ mice were infected with *S. mansoni* and parasites were recovered from the portal tract at 6 weeks post infection. Representative micrographs of parasites recovered from wild type, OT-II/RAG-1^-/-^ and RAG-1^-/-^ mice are shown. Bar = 1 mm. (B) Length of male worms and (C) egg production by schistosome pairs from OT-II/RAG-1^-/-^ (top), DO11.10/RAG-2^-/-^ (center) and Cyt/RAG-1^-/-^ (bottom) mice and their appropriate wild type and RAG^-/-^ controls at 6 weeks post infection. Mean values are represented by horizontal bars. Groups of 4–5 mice were used for each experimental condition. Data are representative of at least two independent experiments.

To test whether TCR specificity for schistosome antigens is necessary for CD4^+^ T cells to facilitate schistosome development, we examined *S. mansoni* development in TCR-transgenic RAG^-/-^ mice that transgenically express previously rearranged MHC class II-restricted TCRs specific for irrelevant antigens. In RAG^-/-^ mice possessing only chicken ovalbumin (OVA)-specific (OT-II/RAG-1^-/-^ C57BL/6, DO11.10/RAG-2^-/-^ BALB/c) or pigeon cytochrome C (PCC)-specific (Cyt5-CC7/RAG-2^-/-^ B10.A) CD4^+^ T cells, schistosome development was enhanced relative to RAG^-/-^ controls that lack CD4^+^ T cells, as determined by assessment of parasite size (Figures 2A and B) and egg production (Figure 2C). The extent to which parasite development was rescued varied between the different strains, with DO11.10/RAG-2^-/-^ mice supporting parasite development that was comparable to wild type mice and parasites from OT-II/RAG-1^-/-^ and Cyt5-CC7/RAG-2^-/-^ exhibiting intermediate levels of development. As expected, OVA- and PCC-specific CD4^+^ T cells from *S. mansoni*-infected RAG^-/-^ mice were completely unresponsive to schistosome antigens (Figures S2A and S2B), though these cells retained the ability to respond to the appropriate antigen (Figure S2B). These data demonstrate that CD4^+^ T cells do not require specificity for schistosome antigens and do not need antigen stimulation in order to facilitate schistosome development. Furthermore, the MHCII molecule by which the TCR is restricted is also likely irrelevant, as the three transgenic TCRs used recognize different murine MHCII molecules. Taken together, the data in Figures 1 and 2 suggest that CD4^+^ T cells influence the outcome of schistosome infection by mechanisms that are independent of antigen receptor specificity and signaling.

### The presence of naïve CD4^+^ cells correlates with steady-state changes in mononuclear phagocyte development

**Figure 3:**
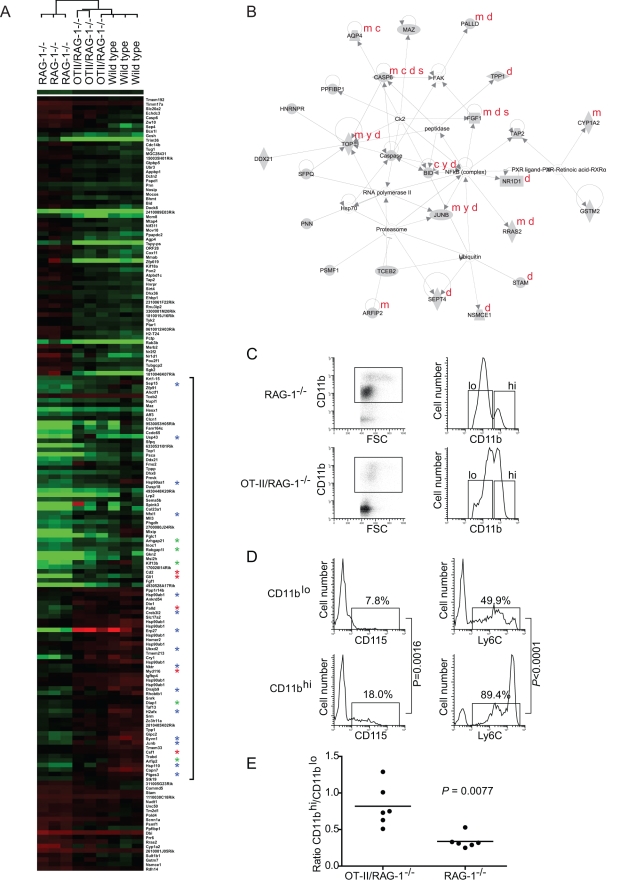
Modulation of gene expression in the liver by naïve CD4^+^ T cells. (A) Microarray analysis revealed 165 genes that are expressed at statistically different (*P*<0.05) levels in liver tissue of both OT-II/RAG-1^-/-^ and wild type mice compared to RAG-1^-/-^ mice. Supervised hierarchical clusters of all 165 differentially expressed genes are shown. Red corresponds to overexpression and green to underexpression, compared to the pooled reference sample. Genes upregulated in OT-II/RAG-1^-/-^ and wild type mice relative to RAG-1^-/-^ mice are indicated by a bracket. Asterisks denote functional annotation as follows: red, myeloid cell development; blue, stress response; green, vesicle formation and transport. For a full list of differentially expressed genes, fold change in expression and statistical significance, see Table S1. Three animals of each genotype were used as a source of RNA for each array. Hybridizations were performed three times (i.e. nine arrays in total). (B) A representative network of functionally related genes from Figure 3A, algorithmically generated based on significant functional gene connectivity in the Ingenuity Pathways Knowledge Base. Network score = 45. Annotations in red denote genes associated with the five most significant biological functions listed in Table S2, as follows: m, Cell Morphology; c, Cellular Compromise; y, Cell Cycle; d, Cell Death; s, Cell-to-Cell Signaling and Interaction. (C) Flow cytometric analysis of peripheral CD11b^+^ mononuclear cells from representative RAG-1^-/-^ (upper panels) and OT-II/RAG-1^-/-^ (lower panels) mice. After excluding dead cells and granulocytes, mononuclear cells were selected based on CD11b positivity and forward scatter (gate, left panels). Cells in this gate resolved into two populations (hi and lo) based on CD11b expression levels (right panels). (D) Analysis of CD115 (left panels) and Ly6C (right panels) expression by CD11b^lo^ (upper panels) and CD11b^hi^ (lower panels) cells defined by the gates in C, right panels. Gates indicate the percentage of gated cells expressing each marker. *P* values were calculated from analysis of 5 mice per group. (E) Relative abundance of CD11b^hi^ and CD11b^lo^ cells in OT-II/RAG-1^-/-^ and RAG-1^-/-^ mice.

Regardless of antigen specificity, naïve CD4^+^ T cells are maintained by active homeostatic processes [12], requiring γ_c_ cytokines and interactions with professional APCs that express MHCII [13], [14], [15], [16], [17], [18]. Because the effect of CD4^+^ T cells on schistosome development is independent of antigen receptor specificity and antigen-mediated activation (Figures 1 and 2), we hypothesized that steady-state homeostatic interactions between naïve CD4^+^ T cells and MHCII^+^ APCs in host tissues may play a role in promoting schistosome development. To identify transcriptional changes induced by the steady-state homeostatic interactions of naïve CD4^+^ T cells, we used a whole genome microarray to compare transcript levels in liver tissue from non-infected RAG-1^-/-^ and OT-II/RAG-1^-/-^ mice (NCBI GEO series accession number GSE8340.) Using this approach, we identified 165 genes that were differentially expressed only in the presence of naïve CD4^+^ T cells (Figure 3A and Table S1). None of these genes are specifically expressed by CD4^+^ T cells, suggesting that OT-II CD4^+^ T cells contribute relatively little to the total hepatic RNA, being relatively few in number and transcriptionally quiescent. Supervised average-linkage hierarchical clustering of the expression data revealed that the transcriptional profile of hepatic tissue from OT-II/RAG-1^-/-^ mice was more similar to that of wild type mice than RAG-1^-/-^ mice, with the single largest grouping of genes comprising those that were expressed at higher levels in wild type and OT-II/RAG-1^-/-^ mice when compared to RAG-1^-/-^ mice (Figure 3A).

To identify biological functions associated with the differentially expressed genes, we employed a structured, knowledge-based approach to identify networks of differentially expressed genes with related functions [19]. Ingenuity Pathways Analysis software (Ingenuity Systems, www.ingenuity.com) was used to overlay differentially expressed genes onto a global molecular network developed from information contained in the Ingenuity Pathways Knowledge Base. Networks of up to 35 differentially expressed genes were then algorithmically generated based on the functional connectivity between the genes. Numerical network scores were calculated to rank networks according to their degree of relevance to the molecules in the dataset. Using this approach, five high-scoring networks (score>20) of differentially expressed genes were identified, a representative of which (score = 45) is illustrated in Figure 3B. Biological functions significantly associated with the networks, together with the genes associated with each function, are displayed in Table S2. Biological functions with the most significant associations included Cell Morphology (*P* = 1.37×10^−4^−4.80×10^−2^), Cellular Compromise (*P* = 1.37×10^−4^−4.31×10^−2^), Cell Cycle (*P* = 2.28×10^−4^−4.58×10^−2^), Cell Death (*P* = 6.30×10^−4^−4.31×10^−2^) and Cell-to-Cell Signaling and Interaction (*P* = 7.51×10^−4^−4.31×10^−2^), suggesting significant changes in cellular development within the liver in the presence of naïve CD4^+^ T cells. Interestingly, genes significantly up-regulated in the presence of naïve CD4^+^ T cells included Csf1/macrophage colony-stimulating factor (M-CSF) (UniGene accession no. Mm.795), which is an essential growth factor for monocytes and monocyte-derived macrophages [20], and other genes involved in myeloid cell development and function (Myd116 (Mm.4048) [21], Cd2 (Mm.22842) [22], Gli1 (Mm.391450) [23] and Plld (Mm.29933) [24]; highlighted in Figure 3A), suggesting specific alterations in mononuclear phagocyte development. Other genes that were up-regulated in the presence of naïve CD4^+^ T cells are associated with the cellular response to stress (highlighted in Figure 3A), including genes involved in the response to mis-folded proteins [25], e.g. several chaperones (Hsp90aa1 (UniGene Accession No. Mm.341186) [26], Hsp90ab1 (Mm.2180) [26], Hsp110 (Mm.270681) [27], p23 (Mm.305816) [28], Dnajb9 (Mm.27432) [29]), enzymes involved in protein folding (Sep15 (Mm.29812) [30], a disulfide isomerase (Mm.33692) [31], a peptidyl-prolyl cis-trans isomerase (Mm.32842)), enzymes involved in protein degradation (Ubxd2 (Mm.29812) [32], the Syvn1 ubiquitin ligase (Mm.149870) [33], Usp43 (Mm.158885)), and DNA-binding proteins involved in the transcriptional response to stress (an XBP-1-like transcription factor (Mm.187453) [34], the ER transmembrane transcription factor Creb3l2 (Mm.391651) [35], H2afx (Mm.245931) [36], and Junb (Mm.1167) [37]). Junb in particular is a central regulator of the cellular response to stress and is targeted for phosphorylation by stress-activated protein kinases [38]. Presumably because of the role of these cells in phagocytosis and in antigen processing and presentation, stress responses are constitutively active in immature APCs and are required for APC development and survival [39], Indeed, the stress response gene Junb is also a critical regulator of myeloid cell differentiation and is classified as a myeloid differentiation primary response (MyD) gene [40]. Also up-regulated in the presence of naïve CD4^+^ T cells were genes involved in another critical aspect of phagocyte and APC function, namely vesicle formation and transport, e.g. Rabgap1l (Mm.25833) [41], Arhgap21 (Mm.28507) [42], Arfip2 (Mm.41637) [43], Kif13b (Mm.23611) [44], and Diap1 (Mm.195916), which is also implicated in receptor-mediated phagocytosis in myeloid cells [45]. Thus, transcriptional changes consistent with alterations in mononuclear phagocyte and/or APC development and function correlate with the presence of naïve CD4^+^ T cells in OT-II/RAG-1^-/-^ mice.

To explore the possibility that mononuclear phagocyte development was altered by the presence of CD4^+^ T cells, leukocytes from the spleen and liver of non-infected RAG-1^-/-^ and OT-II/RAG-1^-/-^ mice were compared by flow cytometry. After gating to exclude granulocytes and OT-II T cells, mononuclear phagocytes were identified by expression of the myeloid marker CD11b (Mm.262106) [46]. In both mouse strains, CD11b^+^ cells segregated into two populations based on relative CD11b expression levels and forward scattering (FSC) properties – CD11b^hi^ FSC^hi^ and CD11b^lo^ FSC^lo^ (Figure 3C). However, the relative proportions of the two populations differed markedly in the two strains, with RAG-1^-/-^ mice possessing relatively more CD11b^lo^ cells than OT-II/RAG-1^-/-^ mice (Figure 3C). Further analysis revealed that the two CD11b^+^ populations also differed significantly in expression of CD115 (Mm.22574, the receptor for M-CSF), with CD11^hi^ cells exhibiting higher CD115 expression than CD11^lo^ cells (Figure 3D; *P* = 0.0016). Both CD11b^+^ populations also expressed Ly6C (Mm.1583, a monocyte marker), although again, CD11b^hi^ cells exhibited higher levels than CD11b^lo^ cells (Figure 3D). Together, the greater forward scatter and elevated expression of CD115 by CD11b^hi^ cells strongly suggest these are functionally more mature mononuclear phagocytes than CD11b^lo^ cells, as increased size and expression of CD11b and CD115 are all implicated in monocyte activation and maturation [47], [48], [49]. Finally, systematic analysis of CD11b^hi^ and CD11b^lo^ populations in age-matched groups of non-infected OT-II/RAG-1^-/-^ and RAG-1^-/-^ mice revealed that cells of the functionally more mature CD11b^hi^ phenotype were significantly more abundant in OT-II/RAG-1^-/-^ mice than RAG-1^-/-^ mice (Figure 3E). Thus, our data suggest that, compared to OT-II/RAG-1^-/-^ mice, RAG-1^-/-^ mice exhibit a defect in mononuclear phagocyte development, with accumulation of cells that possess an immature CD11b^lo^ phenotype. Furthermore, this alteration in myeloid cell development correlates with reduced expression of genes that are required for mononuclear phagocyte development and function (Figure 3A, Figure 3B, Tables S1 and S2). As the only difference between RAG-1^-/-^ and OT-II/RAG-1^-/-^ mice is that, in the latter, transgenic expression of a previously rearranged MHC II-restricted TCR allows for development of a monospecific population of CD4^+^ T cells with specificity for chicken ovalbumin, our data suggest that steady-state MHCII-TCR-mediated interactions between mononuclear cells and naïve CD4^+^ T cells enhances developmental progression of mononuclear phagocytes to a functionally more mature state.

### Direct activation of mononuclear phagocytes rescues schistosome development in the absence of CD4^+^ T cells

**Figure 4:**
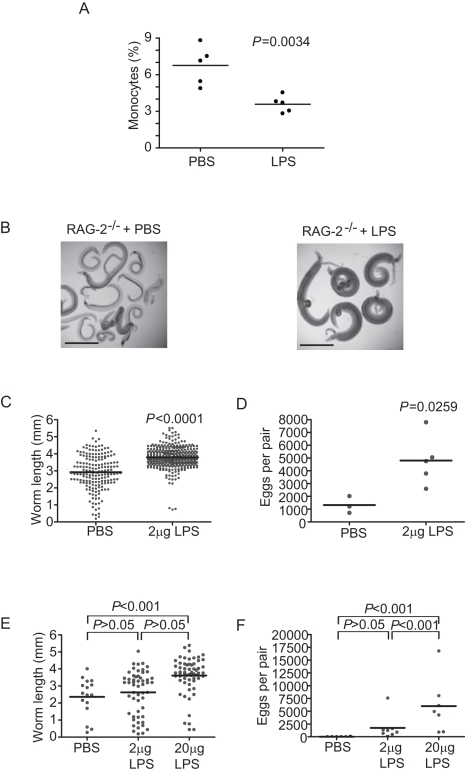
TLR ligand-induced maturation of mononuclear cells facilitates *S. mansoni* development. (A) Relative numbers of monocytes in the spleens of LPS-treated and control (PBS-treated) RAG-1^-/-^ mice. (B) Representative micrographs of parasites isolated from RAG-2^-/-^ mice at six weeks post infection after weekly treatment with 2 µg or LPS. Control animals received carrier (PBS) alone. (C) Length of male worms and (D) egg production by schistosome pairs from RAG-2^-/-^ BALB/c mice at six weeks post infection after weekly treatment with 2 µg or LPS. Control animals received carrier (PBS) alone. Mean values are represented by horizontal bars. (E) Length of male worms and (F) egg production by schistosome pairs from RAG-1^-/-^ C57BL/6 mice at six weeks post infection after weekly treatment with 2 µg or 20 µg LPS. Control animals received carrier (PBS) alone. Mean values are represented by horizontal bars. Groups of 4–5 mice were used for each experimental condition. Data are representative of at least five independent experiments.

Since enhanced parasite development and egg production in OT-II/RAG-1^-/-^ mice correlated with enhanced steady-state maturation of monocytes in these animals, we hypothesized that non-responsive OT-II CD4^+^ T cells facilitate schistosome development indirectly, through steady-state interactions with mononuclear cells that promote monocyte maturation. To test this hypothesis, we tested whether direct stimulation of mononuclear phagocyte maturation, in the absence of any CD4^+^ T cells, could substitute for CD4^+^ T cells in restoring parasite development in RAG^-/-^ mice. As the Toll-like receptor (TLR) -4 (Mm.38049) ligand lipopolysaccharide (LPS) has previously been shown to stimulate the *in vivo* maturation of monocytes [50], [51], we administered ultrapure LPS to schistosome-infected RAG^-/-^ mice during pre-patent infection and assessed the effect on parasite growth and egg production at 6 weeks post infection. Consistent with other reports [50], we found that LPS administration caused a significant decrease in the relative numbers of CD11b^+^ monocytes (Figure 4A), indicative of the terminal maturation and subsequent apoptosis of these cells in response to TLR-4 ligation. Treatment of both BALB/c RAG-2^-/-^ (Figures 4B-D) and C57BL/6 RAG-1^-/-^ (Figures 4E and 4F) mice with LPS led to significant increases in both parasite length (Figures 4B, 4C and 4E) and egg production (Figure 4D and 4F). Indeed, parasite growth (Figure 4E) and egg production (Figure 4F) were responsive to increasing doses of LPS. Together these data indicate that innate immune signals alone are sufficient to support schistosome development and suggest that blood flukes exploit the role of CD4^+^ T cells in providing help for mononuclear phagocyte maturation and function.

## Discussion

Parasites of the genus *Schistosoma* likely arose over 70 million years ago [52] and have undergone complex co-evolution with their definitive hosts, resulting in parasite adaptations that both evade and exploit host immune functions. Previous studies showed that schistosomes require host CD4^+^ T cells for normal development [5], [53], [54] and to mediate egress of eggs from the body across the bowel wall [3], [55]. While egg excretion requires induction of an effector Th2 response to egg antigens [6], the CD4^+^ T cell effector functions that facilitate blood fluke development have not been elucidated. Because previous studies failed to identify a specific role for either Th1 or Th2 responses in schistosome development [5], [8], the question of whether any CD4^+^ T effector functions are required to promote schistosome development remained unresolved. Therefore, the purpose of this study was to test whether antigen receptor signaling and subsequent activation of CD4^+^ T cells are necessary for normal parasite development to proceed. Our results show that, in contrast to the requirement for effector T cell responses to facilitate egg excretion, neither recognition of schistosome antigens nor TCR-mediated activation of CD4^+^ T cells are required for normal parasite development. Our findings suggest that none of the effector functions typically associated with CD4^+^ T cell responses are directly implicated in facilitating schistosome development, and may explain why previous attempts to identify a single T cell factor that modulates schistosome development, using knockout mice deficient in individual cytokines or their receptors [8], have been unsuccessful.

The schistosome requirement for CD4^+^ T cells, but the lack of necessity for traditional T cell effector functions, suggests that the steady-state homeostatic activities of naïve CD4^+^ T cells make the host environment more conducive to schistosome development. While the role of homeostatic interactions with MHCII^+^ cells in maintaining the peripheral CD4^+^ T cell pool is well established, the necessity of these same interactions for efficient APC maturation is being increasingly recognized [18], [56], [57]. In T cell-deficient mice, APC development and function are compromised but can be restored by reconstitution of peripheral T cell populations [18]. Furthermore, it was recently demonstrated that T cell conditioning of APCs for efficient maturation occurs under steady-state conditions before the initiation of T cell responses, in the absence of cognate antigen, and is mediated, at least in part, by the co-stimulatory molecule B7-H1 expressed by naïve T cells [57]. We propose that similar steady-state interactions between naïve OT-II T cells and MHCII^+^ cells in OT-II/RAG-1^-/-^ mice account for the baseline increases in APC-related gene expression and the alterations in mononuclear cell maturation we observed in these mice.

Given the ability of naïve T cells to prime for efficient APC maturation, we hypothesized that exploitation of APC function by schistosomes, rather than of CD4^+^ T cells directly, accounts for the enhanced parasite development we observed in TCR-transgenic RAG^-/-^ mice. While we cannot exclude the possibility that unknown factors elaborated by resting, naïve T cells directly influence schistosome development, our hypothesis provides a parsimonious explanation for why naïve CD4^+^ T cells that do not respond to schistosome infection can still influence parasite development. That schistosome development can be restored in the complete absence of CD4^+^ T cells, through direct maturation of APCs, supports this hypothesis by demonstrating that CD4^+^ T cells are not directly required for parasite development.

As the sentinels of the immune system, circulating monocytes and tissue macrophages and dendritic cells, also known as the mononuclear phagocyte system, specifically express pattern recognition receptors (PRRs), including TLRs, which allow for the detection of invading pathogens [58]. Indeed, monocytes express high levels of TLRs and are the predominant producers of proinflammatory cytokines during endotoxic shock [59]. Thus while other cells in RAG^-/-^ mice express PRRs, the monocytes, macrophages and dendritic cells are the predominant responders to pathogen-associated molecular patterns (PAMPs) such as LPS and are the likely mediators of the effect of LPS on schistosome development. Monocytes, macrophages and dendritic cells are ontologically related, as monocytes are the macrophage and dendritic cell precursors that migrate into the tissues, both during steady-state conditions and during infection. In response to infection, PRR ligation stimulates increased monocyte flux to meet the elevated demand for antigen-presenting and effector cells to combat infection [60]. Our demonstration that LPS administration restores schistosome development is therefore further evidence in support of the hypothesis we propose above, that schistosome development is influenced by innate mononuclear cell function. Indeed, our data point to mononuclear cell function as the common mechanism underpinning the enhancement of schistosome development by both naïve CD4^+^ T cells and LPS. This hypothesis is under further investigation.

Several hypotheses can be proposed to explain how mononuclear phagocytes might influence shistosome development. Recruited to sites of infection and tissue damage, mononuclear cells produce cytokines and chemokines in response to activation that could act as cues for developing schistosomes [51]. Alternatively, the localized release of proinflammatory cytokines by mononuclear cells would be predicted to increase blood flow and vascular permeability, perhaps increasing the supply of host factors required for normal parasite development. At sites of inflammation, monocyte-derived cells also contribute to blood vessel remodeling and angiogenesis, by secreting angiogenic factors and trans-differentiating into endothelial cells [61]. As schistosomes are intravascular parasites and early parasite development occurs within portal venules, vessel remodeling may be required to allow for parasite growth. Yet another possibility is that mononuclear phagocytes may directly damage schistosomes, therefore requiring their local depletion for schistosome development to proceed normally. Activated macrophages and dendritic cells can produce nitric oxide [62], a molecule that is toxic to developing schistosomes [63] and mediates immunity to schistosome infection in animals vaccinated with irradiated cercariae [64]. Priming of APC maturation by CD4^+^ T cells or TLR ligands might allow for more rapid progression of these cells to apoptosis in response to activation. In RAG^-/-^ mice, developmental impairment of APCs might allow for their persistence, constituting a persistent source of nitric oxide and/or other molecules that impair schistosome development. If this is the case, analysis of innate responses to developing schistosomes in RAG^-/-^ mice may identify innate effector mechanisms that can be harnessed to enhance immunity to schistosome infection. These hypotheses are currently being tested.

Our data demonstrate that blood flukes do not respond to CD4^+^ T cells directly, but rather respond to signals that originate from the innate immune system. These findings corroborate previous studies where tumor necrosis factor (TNF; Mm.1293), an innate proinflammatory cytokine, was shown to stimulate parasite egg laying in CD4^+^ T cell-deficient *Prkdc^scid/scid^* mice [65]. While TNF does not appear to stimulate parasite development directly [5], [8], the overlap between the TNF receptor and TLR signaling pathways could account for the ability of both ligands to enhance schistosome development [66], [67]. Our findings also have implications for understanding epidemiological associations between schistosomiasis and other infections [68], such as salmonellosis. While schistosome infection has been implicated in persistence of *Salmonella* infection [69], our data also suggest that proinflammatory stimuli produced in response to bacterial LPS could play a role in exacerbating schistosome infection by supporting parasite development in co-infected individuals. These putative immunological and epidemiological associations are currently under investigation. The possible requirement of schistosomes for proinflammatory signals to support normal development is intriguing, as schistosomes, and helminths in general, are largely unable to stimulate such responses themselves due to a lack of potent TLR ligands [70]. It is therefore tempting to speculate that, under evolutionary pressure to avoid immune detection, schistosomes have lost the ability to stimulate the inflammatory feedback required for their successful development and now rely on other mechanisms to generate these essential inflammatory signals.

In summary, our investigation of the mechanism by which CD4^+^ T cells facilitate schistosome development has revealed that blood flukes require neither CD4^+^ T cell responses nor associated effector functions. Furthermore, our data show that schistosomes do not respond directly to CD4^+^ T cells, as their requirement for these cells can be bypassed completely by direct stimulation of innate immune responses. Indeed, our data suggest that the role of CD4^+^ T cells in facilitating schistosome development may be limited to the provision of non-cognate T cell help for the maturation of MHCII^+^ APCs. We provide two lines of evidence in support of this hypothesis. First, non-responsive, naïve CD4^+^ T cells, which also condition immature APCs to undergo maturation, support improved parasite development. Second, direct stimulation of APC maturation in the absence of CD4^+^ T cells restores schistosome development. These data, together with previous findings that macrophages mediate vaccine-induced immunity to schistosome infection [64], implicate mononuclear cells as central host determinants of the outcome of schistosome infection in the definitive host. A detailed understanding of the interactions between blood flukes and the mononuclear phagocyte system could therefore identify opportunities to modulate mononuclear cell function in ways that impair or prevent the establishment of schistosome infections.

## Materials and Methods

### Ethics statement

All animal studies were performed in accordance with protocols approved by the USUHS Institutional Animal Care and Use Committee.

### Experimental mice

RAG-1^-/-^ mice were purchased from Jackson Laboratory (Bar Harbor, ME) and bred in-house to generate sufficient numbers for experiments. Bcl10^-/-^ mice [11] were kindly provided by Dr. Tak Mak. OT-II mice [71] were the kind gift of Dr. Francis Carbone. Bcl10^-/-^ and OT-II mice, originally with a mixed 129/C57BL/6 background, were backcrossed to the C57BL/6 background. OT-II mice were then bred with RAG-1^-/-^ in house, to generate OTII/RAG-1^-/-^ mice. C57BL/6 (National Cancer Institute, Frederick, MD) and 129× C57BL/6 F1 hybrid wild type mice (Taconic) were used as positive controls, although no differences in parasitological parameters were found in parasites recovered from either wild type strain (data not shown). BALB/cTac-TgN(DO11.10)-Rag2^tm1^ (DO11.10/RAG-2^-/-^) mice [72] and B10.A/AiTac-[Tg]TCRCyt5CC7-I-[KO]-Rag2^tm1^ (Cyt/RAG-2^-/-^) mice [73] were kindly provided by Dr. Dragana Jankovic. RAG-2^-/-^ and wild type mice on the BALB/c and B10.A2 backgrounds (Taconic) were used as controls, respectively.

Mice were infected percutaneously via the tail skin with 150 *S. mansoni* cercariae (Puerto Rican strain) shed from infected *Biomphalaria glabrata* snails [74]. All animal studies were performed in accordance with protocols approved by the USUHS Institutional Animal Care and Use Committee.

### Parasite recovery and measurement of parasitological parameters

Parasites were recovered from the portal system by perfusion [74] 42 days post-infection, immediately fixed in 4% neutral-buffered formaldehyde and photographed using a Nikon Coolpix 4500 4.0 megapixel digital camera connected to a Vistavision trinocular dissecting microscope at 20× magnification. Length of male parasites was determined from digital images using ImageJ software (http://rsb.info.nih.gov/ij). Quantitative analysis of parasite length was performed on male worms as male schistosomes always outnumber females in experimental infections and female growth is significantly influenced by pairing with males [75]. Liver tissue was digested in 0.7% trypsin (50 ml) in phosphate-buffered saline (PBS) for 2–3 hours at 37°C, and eggs were counted under a dissecting microscope. Egg production per schistosome pair was determined by dividing the total number of eggs calculated for each mouse liver by the number of parasite pairs recovered.

### Cell isolation and adoptive transfer

Lymph nodes and spleens from wild type C57BL/6 mice or Bcl10^-/-^ mice were dispersed through a 70-µm nylon strainer. Single cell suspensions were washed and red blood cells were lysed using ACK lysing buffer (Quality Biological, Inc.) Cells were incubated with anti-CD4 (Mm.2209) coated microbeads (Miltenyi Biosciences) and separated using Midi-Macs magnetic columns (Miltenyi Biosciences). Flow cytometric analysis of isolated CD4^+^ T cells routinely demonstrated a purity of ∼99%. 3×10^6^ cells suspended in PBS were transferred into RAG-1^-/-^ mice by intravenous injection into a lateral tail vein. Control recipients received PBS alone. All tissue culture reagents used were free of endotoxin as determined by routine testing. Recipient animals were then infected with cercariae 24 hours post transfer, as described above. To verify the efficacy of adoptive transfers at necropsy, splenocytes from reconstituted RAG-1^-/-^ mice were surface labeled with APC-Cy7-conjugated antibodies to CD4, FITC-conjugated antibodies to CD8 (Mm.1858), APC-conjugated antibodies to TCRβ (Mm.333026), PE-conjugated antibodies to NK1.1 (Mm.6180) and PerCp-Cy5.5-conjugated antibodies to CD19 (Mm.4360) (BD Biosciences) and analyzed using a LSR II Optical Bench flow cytometer with FACSDiva and Winlist software, version 5.0 (Verity Software House). The total number of T cells in each recipient after adoptive transfer was estimated by multiplying the percent of CD4^+^TCRβ^+^ cells by the total number of splenocytes for each mouse.

### Analysis of cytokine production

CD11c^+^ cells were isolated from wild type spleens through incubation of cells suspensions with anti-CD11c (Mm.22378) coated microbeads (Miltenyi Biosciences and separated using Midi-Macs magnetic columns (Miltenyi Biosciences). CD4^+^ cells were isolated from spleens and livers of wild type, Bcl10^-/-^ and OTII/RAG-1^-/-^ mice as described above. CD4^+^ T cells and CD11c^+^ cells, pulsed with 50 µg/ml schistosome worm antigen preparation (SWAP)[76], [77], 5 µg/ml OVA or 1 µg/µl anti-CD3 (Mm.210361) (BD Bioscience), were co-cultured for 72 hours in 96 well plates at a ratio of 5×10^5^ CD4^+^ cells to 5×10^4^ CD11c^+^ cells. Culture supernatants were assessed for IFNγ (Mm.240327) and IL-10 (Mm.874) by ELISA using BD Opt EIA Mouse IFNγ and IL-10 antibody pairs and ELISA reagents (BD Bioscience) and analyzed using a Spectramax M2 Plate reader (Molecular Devices).

### Analysis of cell surface molecule expression

For analysis of T cell activation in cells recovered from spleen and liver, expression of CD44 (Mm.423621), CD62L (Mm.1461), CD69 (Mm.74745), and CD25 (Mm.915) was examined by flow cytometry, after gating on CD4^+^TCRβ^+^NK1.1^-^ cells. Cells isolated from spleens and livers were surface labeled with FITC-conjugated antibodies to CD44 or CD69, PE-conjugated antibodies to TCRβ or NK1.1, PerCp-Cy5.5-conjugated antibodies to NK1.1 or CD4, APC-conjugated antibodies to CD62L, and APC-Cy7-conjugated antibodies to CD4 or CD25, (BD Biosciences). For analysis of mononuclear populations, cells were stained with PE-conjugated anti-CD11b (Mm.262106), PerCP-Cy5.5-conjugated anti-Ly6C (Mm.1583) and APC-conjugated anti-CD115 (Mm.22574). Aqua dead cell stain (Invitrogen) was used to discriminate live and dead cells and granulocytes were excluded based on their high side scatter. All samples were analyzed using a LSR II Optical Bench flow cytometer with FACSDiva (BD Biosciences) and Winlist software, version 5.0 (Verity Software House).

### Microarray

Livers from naïve female wild type C57BL/6, RAG-1^-/-^, and OT-II/RAG-1^-/-^ (N = 9 for each genotype) mice were pooled into groups of three and prepared for cDNA microarray analysis. Briefly, RNA was isolated via RNAzol (Tel-Test, Friendswood, TX) and the RNeasy protocol (Qiagen) and analyzed for purity and concentration on a NanoDrop® ND-1000 Spectrophotometer (Wilmington, DE). cDNA was prepared from two 30 µg aliquots of each pooled sample and labeled with either Cy3 or Cy5 fluorescent probes. One 30 µg aliquot from each pool was used to create a background control pool, while the second aliquot was used as the comparative sample. For further isolation and labeling protocol details please refer to http://www.niaid.nih.gov/dir/services/rtb/microarray/protocols.asp. Samples were hybridized as described by Schaupp [78] to Mmbe custom arrays manufactured by the NIAID Microarray Facility. Further information about these arrays can be found at http://www.ncbi.nlm.nih.gov/geo/query/acc.cgi?acc=GPL1057. Hybridizations were performed in triplicate. Images were scanned by GenePix4000B Scanner (Axon Instruments/Molecular Devices, Sunnyvale, CA) and analyzed using the mAdb program (http://madb.niaid.nih.gov/). Signals were calculated as mean intensity – median background. Data were analyzed using Significance Analysis of Microarrays (SAM), version 2.11 (Stanford University) and student's T tests (EXCEL, with *P* values <0.05 considered significant) to identify only those genes that exhibited differential expression in RAG-1^-/-^ liver tissue compared to both OT-II/RAG-1^-/-^ and wild type liver tissue (i.e. genes differentially expressed in RAG-1^-/-^ liver tissue compared to either OT-II/RAG-1^-/-^ or wild type alone were not considered specific to the presence of naïve CD4^+^ T cells). We applied supervised average-linkage hierarchical clustering on differentially regulated genes, as implemented in the program Cluster version 2.11 (M. Eisen; http://www.microarrays.org/software), separately to both the genes and arrays. The results were analyzed, and figures generated, using TreeView version 1.60 (http://www.microarrays.org/software). Initial gene annotation was performed through GoMiner (http://discover.nci.nih.gov/gominer/). Functional analyses and networks of differentially expressed genes were then generated through the use of Ingenuity Pathways Analysis (Ingenuity® Systems, www.ingenuity.com). To identify networks of differentially expressed genes that were functionally related, Ingenuity Pathways Analysis software was used to overlay differentially expressed genes onto a global molecular network developed from information contained in the Ingenuity Pathways Knowledge Base. To facilitate analysis, networks were limited to a size of up to 35 differentially expressed genes. A numerical network score was then calculated for each network, as described under Statistical Analysis below, to determine the probability of obtaining the same network by random chance.

### LPS treatment

In RAG^-/-^ mice, APC maturation was induced by biweekly intraperitoneal injection of low doses of ultrapure LPS, E. coli 0111:B4 (InvivoGen, San Diego), at doses of 2 µg or 20 µg LPS/mouse. Monocyte numbers were monitored by flow cytometry, as described above.

### Statistical analysis

Because unequal variances were observed among some of the groups analyzed in this study, stringent non-parametric tests were used throughout to test the significance of differences between experimental groups. For two groups, significance of differences between experimental groups was tested using Mann-Whitney tests, and for three groups the significance of differences was tested using Kruskal-Wallis tests followed by Dunns' multiple comparison tests. Statistical analyses were performed with GraphPad Prism Version 4.0 software (GraphPad Software, Inc., San Diego, CA). *P* values of less than 0.05 were considered significant. Experiments were repeated at least twice, with 4–5 animals per group. For microarray data, Functional Analysis of differentially expressed genes was performed using Ingenuity Pathways Analysis (Ingenuity® Systems, www.ingenuity.com) to identify networks of differentially expressed genes that were functionally related. For putative networks, a numerical score was calculated from the hypergeometric distribution of the network using the right-tailed Fisher's Exact Test. The network score is the negative log of the Fisher's Exact Test *P* value. The probability that each biological function assigned to the network is due to chance alone was also tested using Fischer's Exact Test. *P* values of less than 0.05 were considered significant.

### Accession numbers

The gene expression data discussed in this manuscript have been deposited in the NCBI's GEO database and are available under GEO series accession number GSE8340.

## Supporting Information

Table S1 Differential gene expression in liver tissue of RAG-1^-/-^ and OT-II/RAG-1^-/-^ mice.
(0.29 MB DOC)

Table S2 Biological Functions associated with genes that are differentially expressed in liver tissue of RAG-1^-/-^ and OT-II/RAG-1^-/-^ mice.
(0.35 MB DOC)

Figure S1 CD4^+^ T cell activation in response to *S. mansoni* infection is impaired in Bcl10^-/-^ mice. (A) Activation marker expression by hepatic or (B) splenic CD4^+^ T cells was evaluated in response to pre-patent *S. mansoni* infection 28 days post-infection. Surface expression of CD44 and CD62L, CD69, and CD25 on gated CD4^+^TCRβ^+^NK1.1^-^ cells is shown. Numbers represent percentages of the CD4^+^TCRβ^+^NK1.1^-^ population within each gate. (C) CD4^+^ cells were isolated from the spleen and liver of infected or control wild type and Bcl10^-/-^ mice and co-cultured with dendritic cells from non-infected wild type mice pulsed with schistosome worm antigen (SWAP). After 72 hours, culture supernatants were collected and IFNγ and IL-10 were measured by ELISA. Data are represented as mean +/− SEM.
(1.54 MB EPS)

Figure S2 CD4^+^ T cell activation in response to *S. mansoni* antigens is impaired in OT-II/RAG-1^-/-^ mice. (A) Activation marker expression by hepatic and splenic CD4^+^ T cells in wild type and OT-II/RAG-1^-/-^ mice was evaluated in response to pre-patent *S. mansoni* infection 28 days post-infection. Surface expression of CD44 and CD62L on gated CD4^+^TCRβ^+^NK1.1^-^ cells is shown. Numbers represent percentages of the CD4^+^TCRβ^+^NK1.1^-^ population within each gate. (B) CD4^+^ cells were isolated from the liver and spleen of infected or control wild type and OT-II/RAG-1^-/-^ mice and co-cultured with dendritic cells from non-infected wild type mice pulsed with schistosome worm antigen (SWAP) or OVA peptide. After 72 hours, culture supernatants were collected and IFNγ and IL-10 were measured by ELISA. Data are represented as mean +/− SEM.
(1.06 MB EPS)
