# Supplementary material for: Blood Fluke Exploitation of Non-Cognate CD4+ T Cell Help to Facilitate Parasite Development
Source: PLoS Pathog. 2010 Apr 29;6(4):e1000892. doi: 10.1371/journal.ppat.1000892 (PMC2861709; doi:10.1371/journal.ppat.1000892)
Supplement: Table S1 — Differential gene expression in liver tissue of RAG-1-/- and OT-II/RAG-1-/- mice. (0.29 MB DOC) [file ppat.1000892.s001.doc]

**Table 1. Differential gene expression in liver tissue of RAG-1^-/-^ and OT-II/RAG-1^-/-^ mice.**

| **Upregulated in OT-II/RAG-1^-/-^ vs RAG-1^-/-^** | | | | **Downregulated in OT-II/RAG-1^-/-^ vs RAG-1^-/-^** | | | |
| --- | --- | --- | --- | --- | --- | --- | --- |
| Gene name | Unigene accession number | Fold change | *P* value | Gene name | Unigene accession number | Fold change | *P* value |
| 9530053H05Rik | Mm.326532 | 4.8 | 0.037 | Timm17a | Mm.2368 | 4.9 | 0.016 |
| Col23a1 | Mm.154093 | 4.3 | 0.002 | Kif18a | Mm.274086 | 4.0 | 0.016 |
| Fmo2 | Mm.10929 | 4.3 | 0.044 | Tm2d1 | Mm.331719 | 3.9 | 0.042 |
| Lrp2 | Mm.23847 | 4.1 | 0.034 | Tyk2 | Mm.20249 | 3.8 | 0.0004 |
| Tmem33 | Mm.23217 | 4.0 | 0.011 | Appbp1 | Mm.237670 | 3.5 | 0.032 |
| Arhgap21 | Mm.28507 | 3.9 | 0.037 | Hnrpr | Mm.31051 | 3.4 | 0.001 |
| Stk19 | Mm.18845 | 3.6 | 0.045 | Rnft1 | Mm.262707 | 3.3 | 0.006 |
| Clcn1 | Mm.386757 | 3.6 | 0.048 | Trmt6 | Mm.34199 | 3.2 | 0.010 |
| Snrk | Mm.257989 | 3.5 | 0.006 | Nosip | Mm.272139 | 3.1 | 0.009 |
| 4930528A17Rik | Mm.391650 | 3.5 | 0.041 | Rdh14 | Mm.119343 | 3.1 | 0.009 |
| Hsp90ab1 | Mm.2180 | 3.4 | 0.036 | Slc20a2 | Mm.323901 | 3.0 | 0.007 |
| Krt1-15 | Mm.38498 | 3.3 | 0.003 | Ppfibp1 | Mm.103382 | 3.0 | 0.033 |
| Dhx8 | Mm.28186 | 3.3 | 0.024 | Ptar1 | Mm.32215 | 2.9 | 0.002 |
| sep15 | Mm.29812 | 3.3 | 0.032 | Sirt4 | Mm.332616 | 2.9 | 0.005 |
| Sfpq | Mm.257276 | 3.2 | 0.021 | Rras2 | Mm.276572 | 2.9 | 0.012 |
| Srm | Mm.10 | 3.2 | 0.024 | 2410089E03Rik | Mm.164436 | 2.8 | 0.006 |
| Ccdc65 | Mm.26205 | 3.1 | 0.006 | Zfp619 | Mm.333800 | 2.8 | 0.009 |
| Usp43 | Mm.158885 | 3.1 | 0.039 | Nif3l1 | Mm.340248 | 2.8 | 0.020 |
| Spink3 | Mm.272 | 3.0 | 0.007 | Pctp | Mm.5062 | 2.8 | 0.025 |
| Hsp110 | Mm.270681 | 3.0 | 0.033 | Tubgcp2 | Mm.40888 | 2.7 | 0.002 |
| Erp27 | Mm.33692 | 2.9 | 0.002 | Bhmt | Mm.329582 | 2.7 | 0.004 |
| Gli1 | Mm.391450 | 2.9 | 0.003 | Tap2 | Mm.14814 | 2.7 | 0.040 |
| Nmnat3 | Mm.294082 | 2.9 | 0.044 | Tspy-ps | Mm.10106 | 2.6 | 0.002 |
| Fam164c | Mm.158810 | 2.8 | 0.003 | H2-T24 | Mm.14573 | 2.6 | 0.005 |
| Aff3 | Mm.336679 | 2.8 | 0.009 | Dhx36 | Mm.224233 | 2.6 | 0.028 |
| Top1 | Mm.217233 | 2.7 | 0.038 | Dctn2 | Mm.167537 | 2.6 | 0.036 |
| Junb | Mm.1167 | 2.7 | 0.042 | Nudt1 | Mm.118846 | 2.5 | 0.013 |
| Ubxd2 | Mm.293321 | 2.6 | 0.003 | 1500035H01Rik | Mm.278597 | 2.5 | 0.014 |
| Dusp18 | Mm.32588 | 2.5 | 0.003 | ORF28 | Mm.101927 | 2.5 | 0.020 |
| Nfxl1 | Mm.187453 | 2.5 | 0.004 | Ehbp1 | Mm.281732 | 2.5 | 0.025 |
| Cd2 | Mm.22842 | 2.5 | 0.010 | Bid | Mm.235081 | 2.4 | 0.003 |
| Csf1 | Mm.795 | 2.5 | 0.023 | Scnn1a | Mm.144114 | 2.4 | 0.010 |
| Sema5b | Mm.42015 | 2.5 | 0.030 | 1810019J16Rik | Mm.299081 | 2.4 | 0.011 |
| Arfip2 | Mm.41637 | 2.5 | 0.032 | Dbi | Mm.2785 | 2.4 | 0.019 |
| Ptges3 | Mm.305816 | 2.5 | 0.033 | Mov10 | Mm.1597 | 2.4 | 0.019 |
| Hesx1 | Mm.4802 | 2.4 | 0.003 | Tug1 | Mm.471608 | 2.4 | 0.047 |
| Taf13 | Mm.24431 | 2.4 | 0.004 | Stam | Mm.273174 | 2.3 | 0.007 |
| 2700080J24Rik | N/A | 2.4 | 0.004 | Gcsh | Mm.258462 | 2.3 | 0.010 |
| Dio1 | Mm.148342 | 2.3 | 0.016 | Prr6 | Mm.390409 | 2.3 | 0.019 |
| 2810405K02Rik | Mm.41868 | 2.3 | 0.017 | Echdc3 | Mm.38342 | 2.3 | 0.024 |
| Pmvk | Mm.34242 | 2.3 | 0.027 | Msrb2 | Mm.27166 | 2.2 | 0.006 |
| Ankrd54 | Mm.24262 | 2.2 | 0.022 | Gtpbp5 | Mm.219658 | 2.2 | 0.007 |
| Zc3h11a | Mm.392517 | 2.1 | 0.008 | Atpbd1c | Mm.266328 | 2.2 | 0.010 |
| Kif13b | Mm.23611 | 2.1 | 0.011 | Lipt1 | Mm.389100 | 2.2 | 0.011 |
| Gkn2 | Mm.46446 | 2.1 | 0.017 | 2310061F22Rik | Mm.478329 | 2.2 | 0.015 |
| Mll3 | Mm.332268 | 2.1 | 0.018 | Pon2 | Mm.126984 | 2.2 | 0.049 |
| 6330531I01Rik | Mm.383938 | 2.1 | 0.019 | Bcs1l | Mm.358700 | 2.1 | 0.017 |
| Capn7 | Mm.201535 | 2.1 | 0.020 | Cdc14b | Mm.25335 | 2.1 | 0.023 |
| Gipc2 | Mm.97 | 2.1 | 0.023 | Rab3b | Mm.41580 | 2.1 | 0.033 |
| Cry1 | Mm.26237 | 2.1 | 0.025 | Sgk2 | Mm.26462 | 2.1 | 0.041 |
| Dnajb9 | Mm.27432 | 2.1 | 0.026 | sept4 | Mm.2214 | 2.1 | 0.047 |
| Slc17a2 | Mm.24030 | 2.1 | 0.034 | Cyp1a2 | Mm.15537 | 2.1 | 0.049 |
| Fgf1 | Mm.241282 | 2.0 | 0.010 | Trim36 | Mm.277382 | 2.0 | 0.023 |
| Msi2h | Mm.270331 | 2.0 | 0.011 | Casp6 | Mm.281379 | 2.0 | 0.031 |
| Ahctf1 | Mm.128165 | 2.0 | 0.015 | Ppapdc2 | Mm.378757 | 2.0 | 0.032 |
| Inoc1 | Mm.330496 | 2.0 | 0.015 | Pold4 | Mm.32518 | 1.9 | 0.014 |
| Ddx21 | Mm.25264 | 2.0 | 0.016 | Papd1 | Mm.49826 | 1.9 | 0.016 |
| Pqlc1 | Mm.29247 | 2.0 | 0.018 | Nsmce1 | Mm.4467 | 1.9 | 0.017 |
| Tpp1 | Mm.20837 | 2.0 | 0.018 | Pou2f1 | Mm.245261 | 1.9 | 0.019 |
| Diap1 | Mm.195916 | 2.0 | 0.020 | 1810046K07Rik | Mm.75315 | 1.9 | 0.020 |
| Creb3l2 | Mm.391651 | 2.0 | 0.020 | 2610001J05Rik | Mm.29615 | 1.9 | 0.028 |
| H2afx | Mm.245931 | 2.0 | 0.028 | Mtap4 | Mm.217318 | 1.9 | 0.036 |
| Syvn1 | Mm.149870 | 2.0 | 0.032 | Mmab | Mm.105182 | 1.9 | 0.039 |
| Palld | Mm.29933 | 1.9 | 0.016 | Gstm7 | Mm.29640 | 1.9 | 0.043 |
| 1700020I14Rik | Mm.379181 | 1.9 | 0.020 | Nr2f2 | Mm.158143 | 1.9 | 0.049 |
| Maz | Mm.378964 | 1.9 | 0.030 | Commd5 | Mm.291716 | 1.8 | 0.039 |
| Mlxip | Mm.83277 | 1.9 | 0.030 | Mcm8 | Mm.157070 | 1.8 | 0.044 |
| Hsp90aa1 | Mm.341186 | 1.9 | 0.041 | 0610012H03Rik | Mm.46188 | 1.8 | 0.045 |
| Tceb2 | Mm.153758 | 1.8 | 0.021 | Unc50 | Mm.27404 | 1.8 | 0.047 |
| Nupl1 | Mm.258051 | 1.8 | 0.029 | Nr1d1 | Mm.390397 | 1.8 | 0.048 |
| Ppp1r14b | Mm.140 | 1.8 | 0.029 | Rnu3ip2 | Mm.239997 | 1.7 | 0.026 |
| Homer2 | Mm.228 | 1.8 | 0.030 | Pnn | Mm.22347 | 1.7 | 0.028 |
| Zfp91 | Mm.290924 | 1.8 | 0.032 | Tmem192 | Mm.294445 | 1.7 | 0.028 |
| Nktr | Mm.32842 | 1.8 | 0.034 | Dock8 | Mm.102485 | 1.7 | 0.032 |
| Tppp | Mm.39752 | 1.8 | 0.041 | Ubr3 | Mm.314576 | 1.7 | 0.036 |
| Psca | Mm.46395 | 1.8 | 0.043 | Mocos | Mm.28252 | 1.7 | 0.041 |
| Rhobtb1 | Mm.26659 | 1.8 | 0.046 | Cox11 | Mm.151940 | 1.7 | 0.046 |
| Phgdh | Mm.371997 | 1.7 | 0.027 | Zw10 | Mm.24791 | 1.7 | 0.050 |
| Trabd | Mm.284710 | 1.7 | 0.028 | Cideb | Mm.476914 | 1.6 | 0.031 |
| Tmem213 | Mm.387207 | 1.7 | 0.032 | Sult1b1 | Mm.23502 | 1.6 | 0.036 |
| Myd116 | Mm.4048 | 1.7 | 0.044 | Aqp4 | Mm.250786 | 1.6 | 0.041 |
| Rabgap1l | Mm.25833 | 1.6 | 0.044 | Psmf1 | Mm.146984 | 1.6 | 0.042 |
| Igfbp4 | Mm.233799 | 1.6 | 0.049 |  |  |  |  |

**Table 1 Legend.** Relative transcript abundance was determined by microarray analysis (see Figure 3). All genes for which significant changes in gene expression were detected (*P*<0.05) are listed, together with fold change in expression and associated *P* value for each gene.
