# Supplementary material for: Blood Fluke Exploitation of Non-Cognate CD4+ T Cell Help to Facilitate Parasite Development
Source: PLoS Pathog. 2010 Apr 29;6(4):e1000892. doi: 10.1371/journal.ppat.1000892 (PMC2861709; doi:10.1371/journal.ppat.1000892)
Supplement: Table S2 — Biological Functions associated with genes that are differentially expressed in liver tissue of RAG-1-/- and OT-II/RAG-1-/- mice. (0.35 MB DOC) [file ppat.1000892.s002.doc]

**Table 2, part A.** Biological Functions associated with genes that are differentially expressed in liver tissue of RAG-1^-/-^ and OT-II/RAG-1^-/-^ mice.

|  | **Biological Function** | | | | | | | | | | | | | | |
| --- | --- | --- | --- | --- | --- | --- | --- | --- | --- | --- | --- | --- | --- | --- | --- |
| **Gene name** | **CC** | **CD** | **CCS** | **CCy** | **CGP** | **CDev** | **CM** | **CFM** | **CAO** | **MT** | **LM** | **RRR** | **GE** | **PS** | **PTM** |
| *Upregulated OT-II/RAG-1^-/-^ vs. RAG-1^-/-^* | | | | | | | | | | | | | | | |
| Lrp2 |  | ● |  |  |  |  |  |  | ● | ● | ● |  |  |  |  |
| Hsp90ab1 |  | ● |  |  |  |  |  |  |  |  | ● |  |  |  | ● |
| Sfpq |  |  |  |  |  |  |  |  |  |  |  |  | ● |  |  |
| Hsp110 | ● |  |  |  |  |  |  |  |  |  |  |  |  |  |  |
| Gli1 |  | ● | ● |  |  |  | ● | ● |  |  |  | ● | ● |  |  |
| Nmnat3 |  | ● |  |  |  |  |  |  |  |  |  |  |  |  |  |
| Top1 |  | ● |  | ● |  |  | ● |  | ● |  |  | ● |  |  |  |
| Junb |  | ● |  | ● | ● | ● | ● |  | ● |  |  |  | ● |  |  |
| Cd2 |  | ● | ● |  | ● |  | ● |  | ● |  |  | ● | ● |  | ● |
| Csf1 |  | ● | ● | ● | ● | ● | ● | ● | ● | ● | ● | ● | ● |  | ● |
| Arfip2 |  |  |  |  |  |  | ● | ● | ● |  |  |  |  |  |  |
| Ptges3 |  |  |  | ● |  |  |  |  |  |  | ● |  | ● |  |  |
| Hesx1 |  |  |  | ● |  |  |  |  |  |  |  |  |  |  |  |
| Pmvk |  |  |  |  |  |  |  |  |  |  | ● |  |  |  |  |
| Kif13b |  |  |  |  |  |  |  |  | ● |  |  |  |  |  |  |
| Fgf1 |  | ● | ● | ● | ● | ● | ● | ● |  |  | ● | ● |  |  | ● |
| Msi2h |  |  |  |  | ● |  |  |  |  |  |  |  |  |  |  |
| Tpp1 |  | ● |  |  |  |  |  |  |  |  | ● |  |  |  |  |
| Diap1 |  |  |  |  |  |  | ● | ● | ● |  |  |  |  |  |  |
| Creb3l2 |  | ● |  |  |  |  |  |  |  |  |  |  |  |  |  |
| H2afx |  |  |  | ● |  |  |  |  | ● |  |  | ● |  |  |  |
| Syvn1 |  | ● |  |  |  |  |  |  |  |  |  |  |  |  | ● |
| Palld |  | ● |  |  |  |  | ● |  | ● |  |  |  |  |  |  |
| Maz |  |  |  |  |  |  |  |  |  |  |  | ● | ● |  |  |
| Mlxip |  |  |  |  |  |  |  |  |  |  |  |  | ● |  |  |
| Hsp90aa1 | ● | ● |  | ● |  |  | ● |  |  |  |  |  |  |  | ● |
| Tceb2 |  |  |  |  |  |  |  |  |  |  |  |  |  | ● | ● |
| Psca |  | ● |  |  |  |  |  |  |  |  |  |  |  |  |  |
| Phgdh |  |  |  |  |  |  |  |  |  | ● | ● |  |  |  |  |
| Myd116 |  | ● | ● | ● |  |  |  | ● |  |  |  |  |  |  |  |
| Igfbp4 |  | ● |  |  | ● |  |  | ● |  |  | ● | ● |  |  |  |
| *Downregulated OT-II/RAG-1^-/-^ vs. RAG-1^-/-^* | | | | | | | | | | | | | | | |
| Tm2d1 |  | ● |  |  |  |  |  |  |  |  |  |  |  |  |  |
| Tyk2 |  | ● | ● |  |  |  |  |  |  |  |  |  |  |  |  |
| Appbp1 |  | ● |  | ● | ● |  |  |  |  |  |  | ● |  |  | ● |
| Sirt4 |  | ● |  | ● |  |  |  |  |  |  |  | ● |  |  | ● |
| Rras2 |  | ● |  |  |  |  | ● |  |  |  |  | ● |  |  |  |
| Pctp |  |  |  |  |  |  |  |  |  | ● | ● |  |  |  |  |
| Tubgcp2 |  |  |  |  |  |  |  |  |  |  |  |  |  | ● | ● |
| Dhx36 |  |  |  |  |  |  |  |  |  |  |  | ● |  |  |  |
| Dctn2 |  | ● |  | ● |  |  | ● |  | ● |  |  | ● |  |  |  |
| Nudt1 |  | ● |  |  |  |  |  |  |  |  |  | ● |  |  |  |
| Bid | ● | ● |  | ● |  |  | ● |  | ● | ● | ● |  |  |  |  |
| Scnn1a |  |  |  |  |  |  | ● |  |  | ● | ● |  |  |  |  |
| Dbi |  |  |  | ● |  |  |  |  |  |  | ● |  |  |  |  |
| Stam |  | ● |  |  |  | ● |  |  |  |  |  |  |  |  |  |
| Gcsh |  |  |  |  |  |  |  |  |  |  |  |  |  |  | ● |
| Msrb2 |  | ● |  |  |  |  |  |  |  |  |  |  |  |  | ● |
| Atpbd1c |  |  |  | ● |  |  |  |  |  |  |  |  |  |  |  |
| Lipt1 |  |  |  |  |  |  |  |  |  |  | ● |  |  |  | ● |
| Pon2 |  |  |  |  |  |  |  |  |  | ● |  |  |  |  |  |
| Bcs1l |  |  |  |  |  |  |  |  |  |  |  |  |  | ● | ● |
| Rab3b |  |  |  |  |  |  |  |  |  | ● |  |  |  |  |  |
| Cyp1a2 |  |  |  |  |  |  | ● |  |  | ● | ● |  |  | ● |  |
| Casp6 | ● | ● | ● |  |  |  | ● | ● | ● |  |  | ● |  |  |  |
| Pold4 |  |  |  |  |  |  |  |  |  |  |  | ● |  |  |  |
| Nsmce1 |  | ● |  |  |  |  |  |  |  |  |  |  |  |  |  |
| Pou2f1 |  |  |  |  |  |  |  |  |  | ● |  | ● | ● |  |  |
| Mtap4 |  | ● |  | ● |  |  | ● |  | ● |  | ● |  |  |  |  |
| Nr2f2 |  |  |  |  |  |  |  |  |  |  | ● |  | ● |  |  |
| Commd5 |  |  |  | ● |  |  |  |  |  |  |  |  |  |  |  |
| Nr1d1 |  | ● |  |  | ● | ● |  |  |  |  |  |  |  |  |  |
| Pnn |  |  |  |  |  |  |  |  |  | ● |  |  |  |  |  |
| Cox11 |  | ● |  |  |  |  |  |  |  |  |  |  |  | ● | ● |
| Zw10 |  |  |  | ● |  |  |  |  | ● |  |  |  |  | ● | ● |
| Sult1b1 |  |  |  |  |  |  |  |  |  |  |  |  |  |  | ● |
| Aqp4 | ● |  |  |  |  |  | ● |  |  |  |  |  |  |  |  |

**Table 2, part B**. Key to Biological Functions significantly associated with differentially expressed genes in Table 2, part A.

| **abbreviation** | **Biological Function** | ***P* value** |
| --- | --- | --- |
| **CC** | Cellular Compromise | 1.37E-04-4.31E-02 |
| **CD** | Cell Death | 6.3E-04-4.31E-02 |
| **CCS** | Cell-To-Cell Signaling and Interaction | 7.51E-04-4.31E-02 |
| **CCy** | Cell Cycle | 2.28E-04-4.58E-02 |
| **CGP** | Cellular Growth and Proliferation | 1.12E-03-4.31E-02 |
| **CDev** | Cellular Development | 1.84E-03-4.31E-02 |
| **CM** | Cell Morphology | 1.37E-04-4.8E-02 |
| **CFM** | Cellular Function and Maintenance | 7.51E-04-4.31E-02 |
| **CAO** | Cellular Assembly and Organization | 8.77E-03-4.8E-02 |
| **MT** | Molecular Transport | 3.99E-03-4.37E-02 |
| **LM** | Lipid Metabolism | 2.64E-03-4.37E-02 |
| **RRR** | DNA Replication, Recombination, and Repair | 4.76E-03-4.94E-02 |
| **GE** | Gene Expression | 8.77E-03-4.31E-02 |
| **PS** | Protein Synthesis | 2.3E-03-3.46E-02 |
| **PTM** | Post-Translational Modification | 2.3E-03-4.66E-02 |

**Table 2 Legend.** A, Biological Functions that were significantly associated with the dataset of differentially expressed genes were identified using Ingenuity Pathways Analysis software. All Biological Functions for which significant associations (*P*<0.05) were detected are listed, and the differentially expressed genes associated with each function are indicated. B, key to the abbreviations used for each Biological Function in A and the *P* value obtained for each function.
